# Comment on: “Blockade of intercostobrachial nerve by an erector spinae plane block at T2 level”—a reply

**DOI:** 10.1186/s40981-023-00657-1

**Published:** 2023-10-04

**Authors:** Takayuki Yoshida, Tatsuo Nakamoto

**Affiliations:** Department of Anesthesiology, Kansai Medical University Medical Center, 10-15 Fumizono-Cho, Moriguchi City, Osaka, 570-8507 Japan

To the Editor,

We thank Sethuraman for their interest in our case report on intercostobrachial nerve blockade produced by an erector spinae plane (ESP) block [1, 2]. We have carefully read their reflections on our case report and would like to explain our viewpoint.

We cited two articles by Race et al. [3] and Johnson et al. [4] to state that the brachial plexus, including the median brachial and antebrachial cutaneous nerves, can be blocked by supraclavicular and infraclavicular approaches for a brachial plexus block from an anatomical point of view. By referring to these two articles, we did not intend to corroborate that these brachial plexus block techniques spared the intercostobrachial nerve.

We admit that the intercostobrachial nerve may be blocked by an infraclavicular brachial plexus block, considering that the local anesthetic is administered to the compartment, deep to the pectoralis minor muscle, where the intercostobrachial nerve lies. Sethuraman cited a study by Bigeleisen and Wilson [5] to argue for the high probability (approximately 80%) of successful intercostobrachial nerve blockade provided by an infraclavicular brachial plexus block. However, this study may have misinterpreted the medial brachial cutaneous nerve block as an intercostobrachial nerve block. The authors assessed the blockade of the intercostobrachial nerve based on a sensory loss on the “skin distal to the axillary hair patch” [5]. We assume that the sensation on this part would not be exclusively innervated by the intercostobrachial nerve but also by the medial brachial cutaneous nerve [6]. As previously discussed, the infraclavicular approach to the brachial plexus block blocks the medial brachial cutaneous nerve. We believe that the authors should have investigated the sensation in the axilla and lateral chest wall to confirm the involvement of the intercostobrachial nerve, as we did in our case report. Furthermore, in our case, the sensation in the axilla and lateral side of the thoracic wall returned to normal 5.5 h after the block. In contrast, the sensory loss in the other areas lasted longer, and the patient started feeling pain 18.5 h after the block. Therefore, we assume that the sensory loss in the lateral chest wall and the upper arm reflected the consequences of different blocks: the ESP block using 10 ml of local anesthetic and the brachial plexus block using 25 ml of the same local anesthetic composition, respectively. Nevertheless, we agree that we should have tested the area of sensory loss after implementing the brachial plexus block before performing the ESP block to clarify which procedure provided the intercostobrachial nerve blockade.

In selecting the intercostobrachial nerve block techniques, we were concerned about potential hematoma and tissue swelling induced by the ultrasound-guided infiltration on the axilla, in addition to the risk of vessel puncture and consequent compromise of the blood flow to the arteriovenous fistula. These potential complications may affect the ease of the surgical procedure because the axilla is close to the surgical site. We believe that an ESP block has a few disadvantages, such as the excessive use of local anesthetic, additional time to perform, and position-related complications, as stated by Sethuraman. On the other hand, because an ESP block is performed at the proximal site, it can provide intercostobrachial nerve blockade, regardless of its highly variable anatomical properties. Moreover, the main aim of our case report was to introduce ESP block as an alternative to achieve sensory loss in the area innervated by the intercostobrachial nerve. Nevertheless, our article is just a case report; further comparative studies are warranted to confirm the possibility of intercostobrachial nerve involvement by the ESP block.

## Data Availability

Not applicable due to patient privacy concerns.

## Abbreviations

ESP: Erector spinae plane
